# Antibiotic prescription for outpatients with influenza and subsequent hospitalisation: A cohort study using insurance data

**DOI:** 10.1111/irv.13221

**Published:** 2023-11-12

**Authors:** Hiroshi Yokomichi, Mie Mochizuki, Joseph Jonathan Lee, Reiji Kojima, Sayaka Horiuchi, Tadao Ooka, Zentaro Yamagata

**Affiliations:** ^1^ Department of Epidemiology and Environmental Medicine University of Yamanashi Chuo Japan; ^2^ Department of Paediatrics University of Yamanashi Chuo Japan; ^3^ Nuffield Department of Primary Care Health Sciences University of Oxford Oxford UK; ^4^ Department of Health Sciences University of Yamanashi Chuo Japan; ^5^ Department of Emergency Medicine Massachusetts General Hospital Boston Massachusetts USA; ^6^ Centre for Birth Cohort Studies University of Yamanashi Chuo Japan

**Keywords:** antibiotics, antiviral, hospitalisation, influenza, peramivir, pneumonia

## Abstract

**Background:**

Whether prophylactic administration of antibiotics to patients with influenza reduces the hospitalisation risk is unknown. We aimed to examine the association between antibiotic prescription in outpatients with influenza infection and subsequent hospitalisation.

**Methods:**

We conducted a cohort study using health insurance records of Japanese clinic and hospital visits between 2012 and 2016. Participants were outpatients (age, 0–74 years) with confirmed influenza infection who were prescribed anti‐influenza medicine. The primary outcomes were the hospitalisation risk from all causes and pneumonia and the duration of hospitalisation due to pneumonia.

**Results:**

We analysed 903,104 outpatient records with 2469 hospitalisations. The risk of hospitalisation was greater in outpatients prescribed anti‐influenza medicine plus antibiotics (0.31% for all causes and 0.18% for pneumonia) than in those prescribed anti‐influenza medicine only (0.27% and 0.17%, respectively). However, the risk of hospitalisation was significantly lower in patients prescribed peramivir and antibiotics than in those prescribed peramivir only. Patients who received add‐on antibiotics had a significantly longer hospital stay (4.12 days) than those who received anti‐influenza medicine only (3.77 days). In all age groups, the hospitalisation risk from pneumonia tended to be greater in those who received antibiotics than in those prescribed anti‐influenza medicine only. However, among older patients (65–74 years), those provided add‐on antibiotics had an average 5.24‐day shorter hospitalisation due to pneumonia than those provided anti‐influenza medicine only (not significant).

**Conclusions:**

In outpatient cases of influenza, patients who are prescribed antibiotics added to antiviral medicines have a higher risk of hospitalisation and longer duration of hospitalisation due to pneumonia.

## 1 INTRODUCTION

An estimated 290,000 to 600,000 people die annually from influenza infection worldwide.^1^ In the United States, approximately 140,000 to 710,000 people are hospitalised from influenza virus infection annually,^2^ and the hospitalisation rate ranges from 8 to 270 per 100,000 people.^3^ Among paediatric patients, approximately 7% of those with influenza develop lower respiratory tract infection.^4^ In Japan, which has a population of 120 million, approximately 1000 per 100,000 people were hospitalised annually with influenza before the coronavirus pandemic.^5^

Unnecessary prescription of antibiotics leads to bacterial tolerance and is a global problem that results in increased mortality and morbidity from resistant bacterial infections.^6^ Antibiotics are commonly prescribed for viral infections. In an ambulatory department in the United States, 43% of cases of antibiotic prescription had no identifiable basis,^7^ leading to speculation that these cases involved self‐limiting viral infections.

Secondary bacterial pneumonia frequently occurs in influenza virus infection; therefore, the use of prophylactic antibiotics may prevent this pneumonia. A study of outpatient departments in the United Kingdom suggested that antibiotics in influenza infection could also expedite recovery from fever.^8^ A trial of administering co‐amoxiclav (combination of amoxicillin and potassium clavulanate) in high‐risk children with influenza‐like illness did not reduce re‐consultation.^9^ However, these studies were limited by imprecise diagnoses because general practitioners in the United Kingdom use the clinical diagnosis of influenza‐like illness, rather than diagnostic tests for influenza. Although antimicrobial resistance is a serious problem in clinical practice, prophylactic administration of antibiotics could be beneficial if it reduces hospitalisations.

Whether the administration of antibiotics in influenza infection can lessen the frequency or duration of hospitalisation is unknown. In Japan, the use of diagnostics tests for influenza is routine. Therefore, we aimed to examine the potential effect of administering an antibiotic to outpatients with influenza infection on subsequent hospitalisation, using administrative claims data from Japan.

## 2 MATERIALS AND METHODS

### 2.1 Patients and data

We analysed administrative claims data provided by Japan Medical Data Center Ltd. (renamed JMDC in 2018; Tokyo, Japan).^5^, ^10^ The source dataset comprised monthly health insurance claim records (903,104 outpatient records with 2469 hospitalisations) of approximately 3 million employees and their dependents, representing 2.4% of the Japanese population, from January 2012 to December 2016. Insurance claims are issued by consultation; therefore, claims of outpatient and inpatient departments are separate. Within the national health insurance programme in Japan, individuals are allowed to consult physicians at any type of hospital and department, and physicians of any speciality can diagnose influenza using diagnostic tests and prescribe anti‐influenza medications accordingly. The age of patients in the source dataset ranged from 0 to 74 years only. Japanese citizens aged 75 years or older (except for individuals who are on public assistance) are covered by another health insurance programme with lower out‐of‐pocket expenses.^5^

### 2.2 Exposure and outcome

The administrative claims data on patients with influenza included age, prescription medicines, examinations and procedures at outpatient or inpatient departments, and the duration of hospitalisation. Records of patients with influenza were detected using the International Classification of Diseases‐10 category codes J10.0, J10.1 and J10.8. In Japan, physicians almost universally diagnose influenza by point‐of‐care testing, followed by prescription of an anti‐influenza medicine.^5^ Patients were included in the study on the basis of a diagnosis of influenza and prescription of anti‐influenza medicine at an outpatient department, and were divided into two groups: with and without prescription of an antibiotic at the same consultation. Antibiotics were considered to be administered at the same time as anti‐influenza medicines when the date of antibiotic administration was the same as the date of anti‐influenza medicine administration.

Administrative insurance claims are issued monthly in Japan. In this study, patients' hospitalisation data were gathered only if the start date of hospitalisation was in the same calendar month that influenza virus infection was observed at an outpatient department. Subsequent hospitalisation was defined as being when the date of admission was after the date of prescription of outpatient anti‐influenza medicines within the same calendar month. Within the dataset, each patient was identified by an insurance number. This process ensured that, even if a patient was admitted to a hospital that was not associated with the outpatient department where influenza was diagnosed, the hospitalisation would still be detectable. Hospitalisation with pneumonia was detected using the following International Classification of Diseases‐10 codes: J00‐J06, J10‐J18, J20‐J22, J40–46, A15–16, A40.3, A43.0, B05.2, B20.6, B25.0, B37.1, B44.0, B44.1 or B45.0. When an outpatient was hospitalised for 1 day or more, we obtained the duration of hospitalisation measured in days, including stays exceeding 1 month.

Anti‐influenza medicines and antibiotics prescribed at outpatient departments were recorded using the Anatomical Therapeutic Chemical Classification System of codes.^11^ Anti‐influenza medicines included laninamivir, oseltamivir, peramivir and zanamivir. Antibiotics were categorised as cephalosporins, penicillin, other beta‐lactams, macrolides, fluoroquinolones and tetracycline. If an outpatient was prescribed two or more types of antibiotics in the same month, or prescribed two or more types of anti‐influenza medicines in the same month, their data were excluded.

### 2.3 Statistical analysis

We examined the number of inpatients who had been prescribed an anti‐influenza medicine alone, or an anti‐influenza medicine plus an antibiotic, for diagnosed influenza infections. The risk and risk difference of hospitalisations from all causes and pneumonia between patients with and without antibiotics, by prescribed anti‐influenza medicine, were calculated. We also estimated the difference in duration of hospitalisation from pneumonia between each prescribed anti‐influenza medicine versus each anti‐influenza medicine plus an antibiotic. The risk and duration of hospitalisation due to pneumonia and between‐group differences were calculated by age group, ranging from <2 years to 65–74 years. We also calculated the hospitalisation risk and risk difference with and without each antibiotic. Fisher's exact test was used to compare proportions, and *t* tests were used to compare durations. The 95% confidence interval (CI) for the difference in risk was calculated using the Wald method. Statistical analyses were performed using SAS statistical software (version 9.4, SAS Institute, Cary, NC, USA). All reported *p* values were two‐sided, and *p* < 0.05 was considered to indicate a significant difference.

## 3 RESULTS

Table 1 shows the characteristics of outpatients who were prescribed an anti‐influenza medicine only (*n* = 779,787) versus an anti‐influenza medicine plus an antibiotic (*n* = 123,317). In both groups, 44%–45% were female patients. Patients aged 6–12 years were the most likely to be prescribed an antibiotic with anti‐influenza medicine.

**TABLE 1:** Characteristics of outpatients prescribed an anti‐influenza medicine for confirmed influenza infection, with or without an antibiotic.

Influenza cases, *N* (%)	Anti‐influenza medicine only	Anti‐influenza medicine + antibiotic
Total	779,787	123,317
Women	349,520 (45%)	54,692 (44%)
Mean age, years (*SD*)	20.9 (17.2)	22.4 (17.6)
Age, years		
Under 2	19,368 (2%)	2042 (2%)
2–5	114,620 (15%)	16,450 (14%)
6–12	243,203 (31%)	36,082 (29%)
13–18	86,068 (11%)	14,510 (12%)
19–44	216,085 (28%)	35,977 (29%)
45–64	95,476 (13%)	17,098 (14%)
65–74	4335 (0.6%)	1029 (0.8%)

Abbreviation: SD, standard deviation.

Table 2 shows the risk and risk difference in hospitalisation between anti‐influenza medicine versus anti‐influenza medicine plus an antibiotic. The all‐cause hospitalisation risk for any type of anti‐influenza medicine plus an antibiotic was significantly higher than that for any anti‐influenza medicine only. Among anti‐influenza medicines, the all‐cause hospitalisation risk in the oseltamivir + antibiotic or zanamivir + antibiotic group was significantly higher than that in the oseltamivir‐ or zanamivir‐only group. Hospitalisation risks from all causes and pneumonia were significantly lower in the peramivir + antibiotic group than in the peramivir‐only group.

**TABLE 2:** Hospitalisation risks by anti‐influenza medicine prescribed to outpatients, with or without add‐on antibiotic.

Influenza cases	No. of outpatient cases	No. of hospitalisations	No. of pneumonia hospitalisations	Co‐administration proportion, %	Hospitalisation risk, %	Risk of hospitalisation from pneumonia, %
Anti‐influenza medicine only	779,787	2083	1302		0.27	0.17
Anti‐influenza medicine + antibiotic	123,317	386	222	13.7	0.31	0.18
Difference in risk (95% CI)					+0.05 (0.01, 0.08)	+0.01 (−0.01, 0.04)
Laninamivir only	306,509	536	295		0.17	0.10
Laninamivir + antibiotic	49,292	105	54	13.9	0.21	0.11
Risk difference (95% CI)					+0.04 (−0.01, 0.08)	+0.001 (−0.02, 0.04)
Oseltamivir only	320,347	1162	763		0.36	0.24
Oseltamivir + antibiotic	45,253	207	121	12.4	0.46	0.27
Risk difference (95% CI)					+0.09 (0.03, 0.16)	+0.03 (−0.02, 0.08)
Peramivir only	18,423	124	93		0.67	0.50
Peramivir + antibiotic	5163	18	16	21.9	0.35	0.31
Risk difference (95% CI)					−0.32 (−0.52, −0.12)	−0.19 (−0.38, −0.01)
Zanamivir only	134,508	261	151		0.19	0.11
Zanamivir + antibiotic	23,609	56	31	14.9	0.24	0.13
Risk difference (95% CI)					+0.04 (0.03, 0.11)	+0.02 (−0.03, 0.07)

Abbreviation: CI, confidence interval.

Table 3 shows the mean duration of hospitalisation from pneumonia and differences between groups of outpatients treated with each anti‐influenza medicine only versus an anti‐influenza medicine plus an antibiotic. Overall, the anti‐influenza medicine (all) + antibiotic group had a significantly longer hospital stay due to pneumonia than the anti‐influenza medicine only group (*p* = 0.045), but there was no significant difference in the hospitalisation duration from pneumonia with each anti‐influenza medicine.

**TABLE 3:** Hospitalisation duration from pneumonia by type of anti‐influenza medicine prescribed to outpatients with confirmed influenza infection, with or without add‐on antibiotic.

Mean (*SD*)	Hospitalisation duration, days	*p* for difference^a^
All anti‐influenza drugs only (*n* = 533)	3.77 (2.57)	
All anti‐influenza drugs + antibiotic (*n* = 401)	4.12 (2.67)	
Difference^a^ (95% CI)	0.35 (0.007, 0.69)	0.045
Laninamivir only (*n* = 110)	3.89 (2.57)	
Laninamivir + antibiotic (*n* = 95)	4.07 (3.18)	
Difference^a^ (95% CI)	0.18 (−0.65, 1.02)	0.67
Oseltamivir only (*n* = 331)	3.76 (2.57)	
Oseltamivir + antibiotic (*n* = 235)	4.06 (2.31)	
Difference^a^ (95% CI)	0.30 (−0.11, 0.71)	0.15
Peramivir only (*n* = 35)	4.29 (2.42)	
Peramivir + antibiotic (*n* = 23)	4.87 (4.00)	
Difference^a^ (95% CI)	0.58 (−1.31, 2.48)	0.53
Zanamivir only (*n* = 57)	3.26 (2.07)	
Zanamivir + antibiotic (*n* = 48)	4.13 (2.46)	
Difference^a^ (95% CI)	0.86 (−0.03, 1.75)	0.058

Abbreviations: CI, confidence interval; SD, standard deviation.

^a^ Difference in duration of hospitalisation from pneumonia between the antiviral only and the antiviral + antibiotic groups.

Table 4 shows the risk and duration of hospitalisation due to pneumonia in patients treated with anti‐influenza medicine only versus anti‐influenza medicine plus an antibiotic, by age group. Anti‐influenza medicine plus an antibiotic were significantly associated with a greater risk of hospitalisation due to pneumonia among patients in four age groups: 2–5 years (0.57%), 6–12 years (0.22%), 19–44 years (0.16%) and 45–64 years (0.33%). The mean duration of hospitalisation due to pneumonia was also significantly longer by 0.61 days (95% CI [0.16, 1.06]) in the age group of 2–5 years who received anti‐influenza medicine plus an antibiotic compared with those who received anti‐influenza medicine only. In contrast, in the older age group (65–74 years), the mean duration of hospital stay was shorter by 5.24 days (95% CI [−22.2, 11.7]) among those who received anti‐influenza medicine plus an antibiotic compared with those who received anti‐influenza medicine only, but this was not significant.

**TABLE 4:** Risk and mean duration of hospitalisation with pneumonia in those prescribed anti‐influenza medicine, with or without add‐on antibiotic, by age group.

Age group, years	Under 2	2–5	6–12	13–18	19–44	45–64	65–74
Pneumonia hospitalisation risk							
Anti‐influenza med only (A), %	3.71	0.83	0.38	0.21	0.13	0.24	1.1
Anti‐influenza med + antibiotic (B), %	3.73	1.40	0.61	0.30	0.29	0.56	3.0
Difference (B–A), %	0.02 (−1.4, 1.4)	0.57 (0.29, 0.85)	0.22 (0.08, 0.36)	0.10 (−0.007, 0.27)	0.16 (0.04, 0.28)	0.33 (0.02, 0.63)	1.9 (−1.0, 4.8)
Hospitalisation duration from pneumonia							
Anti‐influenza med only (C), days	4.19	3.21	3.18	4.27	4.90	7.30	11.67
Anti‐influenza med + antibiotic (D), days	3.87	3.82	3.60	3.85	5.41	6.91	6.43
Difference (D–C), days	−0.33 (−1.09, 0.44)	0.61 (0.16, 1.06)	0.42 (−0.04, 0.88)	−0.42 (−1.67, 0.84)	0.51 (−1.00, 2.02)	−0.39 (−3.41, 2.62)	−5.24 (−22.2, 11.7)

Table 5 shows the risks and risk differences in hospitalisation in outpatients prescribed anti‐influenza medicine plus different classes of antibiotics compared with anti‐influenza medicine alone. Risk differences in hospitalisation due to all causes and pneumonia were significantly greater in patients prescribed an anti‐influenza medicine plus any antibiotic. Among those who received an antibiotic, the hospitalisation risk was lowest in the macrolide group (0.30% for hospitalisation due to all causes and 0.24% for hospitalisation due to pneumonia).

**TABLE 5:** Hospitalisation risk in outpatients prescribed anti‐influenza medicine with add‐on antibiotic, categorised by antibiotic class.

Influenza cases	Hospitalisation risk, %	Risk difference, % (95% CI)	Pneumonia hospitalisation risk, %	Risk difference, % (95% CI)
No antibiotics	0.22	―	0.16	―
Cephalosporin	0.35	0.13 [0.08, 0.17]	0.28	0.12 [0.08, 0.16]
Penicillin	0.62	0.39 [0.26, 0.53]	0.46	0.30 [0.18, 0.41]
Other beta‐lactam	0.69	0.47 [0.14, 0.79]	0.65	0.49 [0.17, 0.81]
Macrolide	0.30	0.08 [0.04, 0.11]	0.24	0.08 [0.05, 0.11]
Fluoroquinolone	0.40	0.18 [0.10, 0.26]	0.29	0.13 [0.06, 0.20]
Tetracycline series	0.72	0.50 [0.15, 0.86]	0.63	0.48 [0.14, 0.81]

Abbreviation: CI, confidence interval.

## 4 DISCUSSION

Our data showed that the hospitalisation risk from all causes was significantly higher in outpatients prescribed with anti‐influenza medicine plus an antibiotic than in those prescribed with anti‐influenza medicine only (Table 2). In patients who received peramivir, the hospitalisation risk was significantly lower when an antibiotic was added, whereas those who received oseltamivir or zanamivir plus an antibiotic had a significantly higher hospitalisation risk than those with no antibiotic. The hospitalisation risk was not reduced by prescription of any class of antibiotics (Table 5). Among the antibiotics analysed, macrolides were associated with the smallest hospitalisation risk. Overall, the duration of hospitalisation from pneumonia was longer with each anti‐influenza medicine plus an antibiotic, but this was not significant (Table 3). One exception to this finding was in the older age (65–74 years) group. In the older age group, hospitalisation due to pneumonia appeared to be shorter by 5.24 days in those who received an add‐on of antibiotics than in those who received anti‐influenza medicine only, but the number of patients was small and this result was not significant (Table 4).

The dangers of prophylactic administration of antibiotics have been recognised among health professionals for many years, mainly because it can lead to the generation of resistant strains.^12^ In our study, patients with add‐on antibiotics had a higher risk of hospitalisation due to all causes (Table 2), which suggested that physicians prescribed antibiotics when an outpatient's symptoms were severe. Indeed, the mean duration of hospital stay due to pneumonia was longer in those who received add‐on antibiotics (Table 3).

Antibiotics are ineffective against viral infections. However, infection with influenza has a higher hospital admission risk than many other respiratory viruses and is associated with secondary bacterial infections.^13^ A randomised, controlled trial concluded that early use of antibiotics in children with influenza‐like illness did not reduce the risk of re‐consultation, but did reduce symptoms of the respiratory disease.^9^ This finding is in line with our data, which suggest that antibiotics do not decrease the hospitalisation risk from all causes or pneumonia in cases of true influenza infection.

Although adding an antibiotic to peramivir was associated with a significantly decreased hospitalisation risk from all causes and pneumonia, it did not affect the duration of hospitalisation from pneumonia (Tables 2 and 3). Peramivir is delivered via intravenous infusion. Therefore, peramivir might have been administered to more severely ill outpatients who were unable to inhale medicines or eat or drink. The literature suggests that peramivir tends to reduce the time to alleviation of influenza symptoms.^14^ Antibiotics also might have been administered intravenously in these patients in our study. Our further analysis showed that 64.8% of peramivir users were patients aged 19–64 years, and this percentage was greater than that of the other anti‐influenza medicine users. Moreover, 21.9% of peramivir users used antibiotics simultaneously. This percentage was also greater than that of the other anti‐influenza medicine users. These findings suggested that the combination of peramivir and antibiotics at outpatient departments was administered mainly to adults, and this prescription may have decreased hospitalisation in this age group.

Children younger than 2 years are vulnerable to bacterial infections because they have immature immune systems and may not have been vaccinated against haemophilus influenzae, pneumococcus, diphtheria and other pathogens.^15^ This fact may explain why adding antibiotics appeared to be associated with a lower risk difference for hospitalisation due to pneumonia in the comparison between anti‐influenza medicine versus anti‐influenza medicine plus an antibiotic in this age group (+0.02, Table 4). Additionally, when hospital admission was restricted to pneumonia, add‐on antibiotics may have reduced the hospitalisation duration in children younger than 2 years (Table 4).

Patients older than 65 years are also vulnerable to bacterial infections. Pneumonia is the fourth highest cause of death in this age group in Japan. In UK real‐world data, the rate of pneumonia after chest infections was reduced from 403 per 10,000 common respiratory infections to 146 per 10,000 when the physician prescribed antibiotics.^16^ Pneumococcal vaccination is optional in Japan; therefore, coverage is not high.^17^ Adding a prophylactic antibiotic may have protected older patients from secondary bacterial infections because it appeared to be associated with 5.24 fewer days of hospitalisation (not significant) from pneumonia in the age group of 65–74 years (Table 4).

Among the antibiotics, patients treated with macrolide antibiotics were the least likely to be hospitalised for all causes and pneumonia (Table 5). Macrolides have a broad spectrum of activity against pathogenic microbes, including staphylococci, mycoplasma and chlamydia. The effect of these antibiotics is bacteriostatic, and they increase ciliary movement in the airway mucosa.^18^ Although macrolide‐resistant bacteria are problematic worldwide,^19^ prophylaxis with macrolides may be a reasonable choice in outpatient departments if there is an indication because they may be associated with decreased hospitalisation.

Overall, our results support refraining from the prophylactic prescription of antibiotics in influenza. Although prophylactic administration is not recommended for any age group, adding an antibiotic to an anti‐influenza medicine might be considered in an older patient with influenza who has not been vaccinated for pneumococcus or has an underlying disease.

The observational nature of this study limits the interpretation of the effects of add‐on antibiotics for patients with influenza infection. Randomised, controlled studies could enable estimation of the risks and benefits of antibiotic administration. We presume that physicians tend to prescribe add‐on antibiotics to patients with influenza who have severe symptoms, and this confounding by indication may have limited our study. Additionally, understanding what leads physicians to prescribe antibiotics in different types of patients, how physicians develop an intuitive sense that they should prescribe antibiotics in the absence of confirmed bacterial infection, and the accuracy of such decision‐making is important. Observational studies are required to answer these questions.

Another limitation of this study is that we did not have data regarding co‐infection of influenza virus and bacteria. A recent meta‐analysis showed that 23% of patients hospitalised with laboratory‐confirmed influenza had co‐infection of bacteria.^20^ The reported percentages in this meta‐analysis ranged widely from 1.9% to 65.4%, and varied by situation and country. The meta‐analysis did not include a Japanese study, and to the best of our knowledge, there are no available data on bacterial co‐infection of outpatients with influenza in Japan. Our results support the further analysis of real‐world data regarding co‐infection of influenza virus and bacteria, prescription of anti‐influenza medicine with and without antibiotics, and hospitalisation.

This study has some other limitations. The results were limited to the data of employees and their families, and did not include business owners, freelancers or adults aged >75 years. We also chose not to analyse overlapping prescriptions of more than one antibiotic because of the concern that overlapping data would confuse the results. Moreover, in Japan, almost all anti‐influenza medicines are prescribed after confirmation of influenza cases by point‐of‐care testing. However, there are rare cases when anti‐influenza medicines may be prescribed to patients with a high fever because they are known to have influenza because a family member has influenza. In addition, in Japan, prophylactic administration of anti‐influenza medications for close contact with patients with influenza is not permitted under the insurance scheme. Finally, because the add‐on antibiotics that were identified were those prescribed in outpatient departments, switching of antibiotics in hospitalised patients was not analysed.

There are some strengths of this study. First, we included a large number of analysed patients. Second, the results were from real‐world data of administrative health insurance claims. Third, the diagnosis of influenza was based on point‐of‐care testing.^5^ In Japan, data on influenza and influenza‐like illness are separated with much greater precision than countries where testing is not as widespread. Fourth, we were able to stratify the results with several anti‐influenza medicines, which are commonly used in Japan. Fifth, the study period occurred well before the start of the coronavirus pandemic and infection control measures; therefore, the results were reflective of a normal influenza epidemic.

In conclusion, our administrative data indicated that outpatients prescribed an anti‐influenza medicine plus an antibiotic had a higher risk of hospitalisation and longer duration of hospitalisation due to pneumonia. In patients aged 65–74 years, administration of add‐on antibiotics at outpatient departments was associated with a reduced hospital duration, albeit this was not significant. Among the anti‐influenza medicines, there were lower risks of hospitalisation due to all causes and pneumonia in patients who received peramivir with an antibiotic. Among the antibiotics, the lowest hospitalisation risk was in patients who were prescribed macrolides.

## AUTHOR CONTRIBUTIONS

**Hiroshi Yokomichi:** Conceptualization (lead); data curation (equal); formal analysis (equal); funding acquisition (equal); project administration (equal); writing—original draft (equal); writing—review and editing (equal). **Mie Mochizuki:** Conceptualization (equal); methodology (lead); writing—original draft (equal); writing—review and editing (equal). **Joseph Jonathan Lee:** Funding acquisition (supporting); writing—original draft (equal); writing—review and editing (equal). **Reiji Kojima:** Writing—original draft (supporting); writing—review and editing (supporting). **Sayaka Horiuchi:** Writing—original draft (supporting); writing—review and editing (supporting). **Tadao Ooka:** Writing—original draft (supporting); writing—review and editing (supporting). **Zentaro Yamagata:** Writing—original draft (supporting); writing—review and editing (supporting).

## CONFLICT OF INTEREST STATEMENT

The authors have no competing interests to declare.

### PEER REVIEW

The peer review history for this article is available at https://www.webofscience.com/api/gateway/wos/peer-review/10.1111/irv.13221.

## ETHICS STATEMENT

The ethics committee of the School of Medicine, University of Yamanashi, approved this study (approval number H29‐1709), in accordance with the ethical guidelines and regulations of the Declaration of Helsinki. The data were properly anonymised by the JMDC in the manner permitted by the Japanese Guideline of Personal Information Protection Commission, Cabinet Office, Government of Japan, for the use of data from medical examinations in medical research without the consent of individual participants.

## Data Availability

The original administrative data are available through a formal request to the JMDC, subject to fees.
